# Migrating eastern North Pacific gray whale call and blow rates estimated from acoustic recordings, infrared camera video, and visual sightings

**DOI:** 10.1038/s41598-019-49115-y

**Published:** 2019-08-30

**Authors:** Regina A. Guazzo, David W. Weller, Hollis M. Europe, John W. Durban, Gerald L. D’Spain, John A. Hildebrand

**Affiliations:** 1 Scripps Institution of Oceanography, University of California San Diego, La Jolla, California 92093-0205 United States of America; 2 Naval Information Warfare Center Pacific, San Diego, California 92152-5001 United States of America; 3 National Oceanic and Atmospheric Administration, Southwest Fisheries Science Center, La Jolla, California 92037-1508 United States of America

**Keywords:** Animal migration, Behavioural ecology, Marine biology

## Abstract

During the eastern North Pacific gray whale 2014–2015 southbound migration, acoustic call recordings, infrared blow detections, and visual sightings were combined to estimate cue rates, needed to convert detections into abundance. The gray whale acoustic call rate ranged from 2.3–24 calls/whale/day during the peak of the southbound migration with an average of 7.5 calls/whale/day over both the southbound and northbound migrations. The average daily calling rate increased between 30 December–13 February. With a call rate model, we estimated that 4,340 gray whales migrated south before visual observations began on 30 December, which is 2,829 more gray whales than used in the visual estimate, and would add approximately 10% to the abundance estimate. We suggest that visual observers increase their survey effort to all of December to document gray whale presence. The infrared camera blow rate averaged 49 blows/whale/hour over 5–8 January. Probability of detection of a whale blow by the infrared camera was the same at night as during the day. However, probability of detection decreased beyond 2.1 km offshore, whereas visual sightings revealed consistent whale densities up to 3 km offshore. We suggest that future infrared camera surveys use multiple cameras optimised for different ranges offshore.

## Introduction

Estimating population sizes of marine mammals is a challenging problem. Traditionally population size has been calculated using visual surveys including aerial and ship-based line transect surveys and shore-based surveys. However, these types of surveys are time intensive and expensive. Therefore, scientists have dedicated much recent effort to counting animals using autonomous techniques. These methods count cues emitted by the animals and then convert these cues into total abundance. For cetaceans, useful cues are acoustic calls and visible exhalations or blows, but in order to convert these cues into abundance, an accurate cue production rate (or simply, cue rate) is needed.

Eastern North Pacific gray whales (*Eschrichtius robustus* Lilljeborg) annually migrate between summer feeding areas primarily in the Bering and Chukchi Seas and wintering areas in the lagoons of the Baja California Peninsula in Mexico. While migrating, gray whales tend to swim over the continental shelf, and in many places along the west coast of North America these whales can be seen from land. The abundance of the eastern North Pacific population of gray whales has been estimated using shore-based visual surveys during the southbound migration since the 1967–1968 migration^1^. Marine mammal observers from the National Oceanic and Atmospheric Administration (NOAA) count whales from a vantage point approximately 22 m above sea level as the whales pass through a pre-defined study area at Granite Canyon in central California where the continental shelf is narrow. Even though gray whales travel along a very nearshore route in this area, observation and counting periods used to estimate their abundance are limited to daytime periods with adequate visibility and environmental conditions and days when trained visual observers are available. After accounting for probability of detection and amount of time with effort, NOAA estimated the eastern North Pacific gray whale population size to be 28,790 individuals during the 2014–2015 season^2^.

Many marine animals use sound as their primary method of communication and sensing their environment. Odontocetes, or toothed whales, use whistles and/or clicks to communicate and echolocation clicks to sense their environment and detect prey. Mysticetes, or baleen whales, make low- and mid-frequency vocalizations to communicate across vast distances. Passive acoustic monitoring is commonly used to determine presence of a vocalizing species as these calls are often species-specific. Marques *et al*.^3^ showed how the number of animals could be estimated from the number of calls. A required variable in this calculation is the calling rate of the species, which may change over time, location, age, sex, and/or with behavioural state. For example, migrating humpback whales off the east coast of Australia have decreased their singing rate as the population size has increased over 18 years^4^. Bryde’s whales call at different rates depending on their geographic region with whales in the Eastern Tropical Pacific producing calls at a slower rate (intercall interval of 0.3–5.5 min) than those in Japan (intercall interval of 0.05–0.29 min)^5^. These studies exemplify the importance of estimating calling rate for the time and location of interest.

Gray whales produce M3 calls as their primary call type while migrating^6,7^. M3 calls are short, low-frequency calls with a mean duration of 1.8 s and a mean frequency of 48 Hz^8^. Gray whale M3 calls are produced at a lower source level than many other baleen whale vocalizations and have an average root mean square (RMS) source level of 157 dB re l*μ*Pa at 1 m^8^. Dalheim^9^ recorded gray whales calls in the Baja California lagoons and reported calling rates for all call types of 0.33 calls/whale/hour (7.92 calls/whale/day) in February and 0.25 calls/whale/hour (6 calls/whale/day) in March. Gray whales were thought to produce fewer calls while migrating than while in their breeding areas^6,7^, but Guazzo *et al*.^8^ estimated that 4,854 calls were produced within a search area offshore of the NOAA visual observation site, resulting in an average calling rate of 5.7 calls/whale/day.

Since whales must surface to breathe and their blows are slightly warmer than the surrounding water^10^, infrared cameras are another method for monitoring whales. These cameras are capable of detecting whale exhalations (hereafter called blows) both at night and during the day. Even though the core body temperature of baleen whales is around 36 °C^11^, the blow of a gray whale is composed mostly of seawater that is entrained in the air due to a subsurface exhalation^12^, resulting in a small temperature difference between the blow and the seawater. In Arctic waters, the mean temperature of whale blows was estimated to be 0.2–3.0 °C above the water temperature^10^. The angle of a target to the camera as well as solar energy scattered off a target can also cause a target, including a whale blow, to appear warmer on infrared images^13^. Sumich^14^ visually observed 74 migrating gray whale groups for at least 15 minutes each and reported an average blow rate of 0.72 blows/whale/min (43.2 blows/whale/hour) with a series of short dives marked by 2–4 blows 20–30 seconds apart followed by a longer dive lasting 3–4 minutes. The range in blow rates was 0.45–1.12 blows/whale/min (27–67.2 blows/whale/hour). Perryman *et al*.^15^ used infrared cameras to study gray whale migration behaviour during the day and night for three different migration seasons. They found no difference in surfacing interval between day and night and reported an average surfacing interval for single animals tracked using infrared cameras of 27 s (133 blows/whale/hour)^15^, which is much shorter than those reported by Sumich^14^. However, in order for Perryman *et al*.^15^ to measure the time intervals between surfacings, a single animal had to blow more than once in the narrow camera field of view. Therefore, their results pertain only to the sequence of short-duration dives and the reported surfacing interval of 27 s is consistent with the 20–30 s interval reported by Sumich^14^. A benefit of thermal sensing over acoustic sensing is that whales are required to breathe while vocalization patterns are more strongly affected by behavioural state. That said, infrared cameras are sensitive to environmental parameters and factors such as humidity, sea state, and glare from the sun can reduce the probability of detection of whale blows^13^.

The objective of the present study was to estimate acoustic and infrared cue rates of migrating gray whales for use in estimating whale abundance in future population censuses. We combine the number of calls and blows detected with the number of gray whales estimated from a visual sighting census. We apply the gray whale calling rate to estimate the number of whales that migrated through the study area prior to the date when visual observations began.

## Results

**Figure 1:**
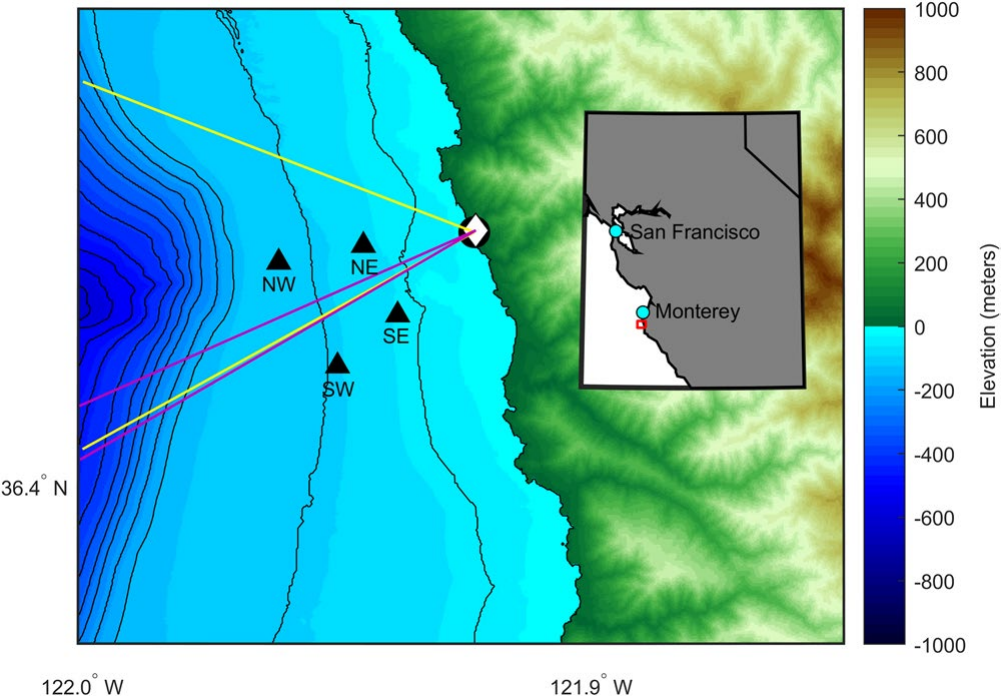
Map of the study area for the combined visual, infrared, and acoustic survey of migrating gray whales. The black circle indicates the location of the visual observers and the white diamond indicates the location of the infrared camera. The four black triangles indicate the locations of the four hydrophones and are labelled according to their positions. The visual field of view is shown with yellow lines and the infrared field of view is shown with pink lines. Colours indicate the land elevation and seafloor depth with respect to sea level and the black contour lines show the seafloor depth in 50 m increments. Bathymetry data are from the NOAA National Centers for Environmental Information’s Southern California Coastal Relief Model with 1 arc-second resolution. The latitude bounds are 36.375°–36.475°N and the longitude bounds are 122.000°–121.850°W. For reference, the SE hydrophone is about 1.5 km from shore and the NW hydrophone is about 3.6 km from shore.

**Figure 2:**
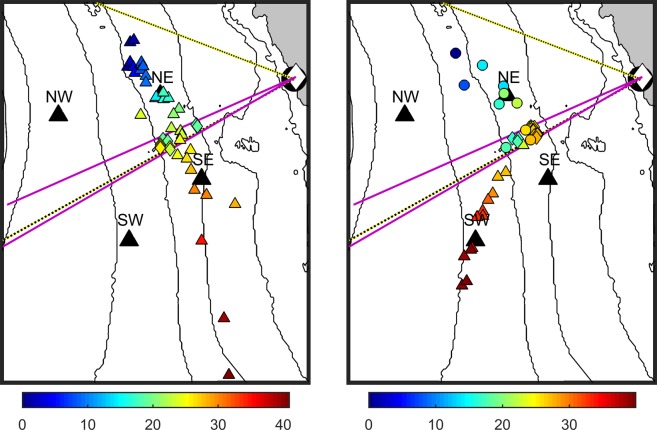
Example gray whale tracks from visual, acoustic, and infrared localizations. Visual sightings are shown with coloured circles, acoustic calls are shown with coloured triangles, and infrared blows are shown with coloured diamonds. Colour indicates the amount of time in minutes since the start of the detections. The left plot spans 06 January 2015 02:14:15–02:55:10 local time (at night so no circles for visual sighting locations are plotted) and the right plot spans 07 January 2015 08:31:01–09:10:58 local time. Four black triangles indicate the positions of hydrophones, the black circle indicates the location of the visual observers, and the white diamond indicates the location of the infrared camera. The visual field of view is shown with yellow lines (with black dotting) and the infrared field of view is shown with pink lines. The black contour lines show the seafloor depth in 20 m intervals (from the NOAA National Centers for Environmental Information’s Southern California Coastal Relief Model with 1 arc-second resolution). Latitude limits are 36.40°–36.45°N and longitude limits are 121.92°–121.97°W.

Southbound migrating eastern North Pacific gray whales were successfully detected and localised using visual sightings, acoustic recordings, and infrared camera video during the 2014–2015 migration season (Fig. 1). Visual observers counted 2,951 southbound migrating gray whales during daylight hours with good visibility on 34 days and estimated the population size to be 28,790 (95% Credibility Interval: 23, 620–39, 210)^2^. Guazzo *et al*.^8^ verified and localised 4,247 gray whale M3 calls within the bounds of the hydrophone array over the entire (southbound and northbound) 2014–2015 migration season. Using probability of detection, they estimated that 4,854 M3 calls were actually produced within the hydrophone array^8^. These calls are unique to gray whales, so we can be certain we are only including gray whales in our calling rate analysis. During the four days (5–8 January 2015) that blows were manually detected on the infrared camera recordings, 1,882 gray whale blows were identified. Visual observers confirmed that no substantial numbers of other species were in the area that could be confused with gray whales on the infrared system. Figure 2 shows two example times during this four-day period of overlap in data processing of sensing system recordings when we were able to detect the same whales using multiple methods.

### Acoustic recordings and visual sightings

By using visual sightings, we were able to estimate with more accuracy the proportion of gray whales that migrated through the area bounded by the hydrophone array than by using the more general results from aerial surveys presented by Shelden and Laake^16^. In an earlier publication, Guazzo *et al*.^8^ estimated that only 10% of gray whales traveled past Granite Canyon outside of the array, but using visual sightings we found that the proportion of whales swimming outside the array was greater than assumed, decreasing the proportion that migrated through the hydrophone array. We updated our 90% estimate^8^, which was based on coarse aerial sightings data from previous migration seasons, and, using more precise visual sightings data from this migration season, were able to estimate that about 68% of whales migrated within the area bounded by the hydrophone array. This decrease in number of animals within the array increases the average calling rate over the entire migration season from 5.7 calls/whale/day^8^ to 7.5 calls/whale/day (0.31 calls/whale/hour) (using the 95% Credibility Interval for gray whale abundance: 5.5–9.1 calls/whale/day).

**Figure 3:**
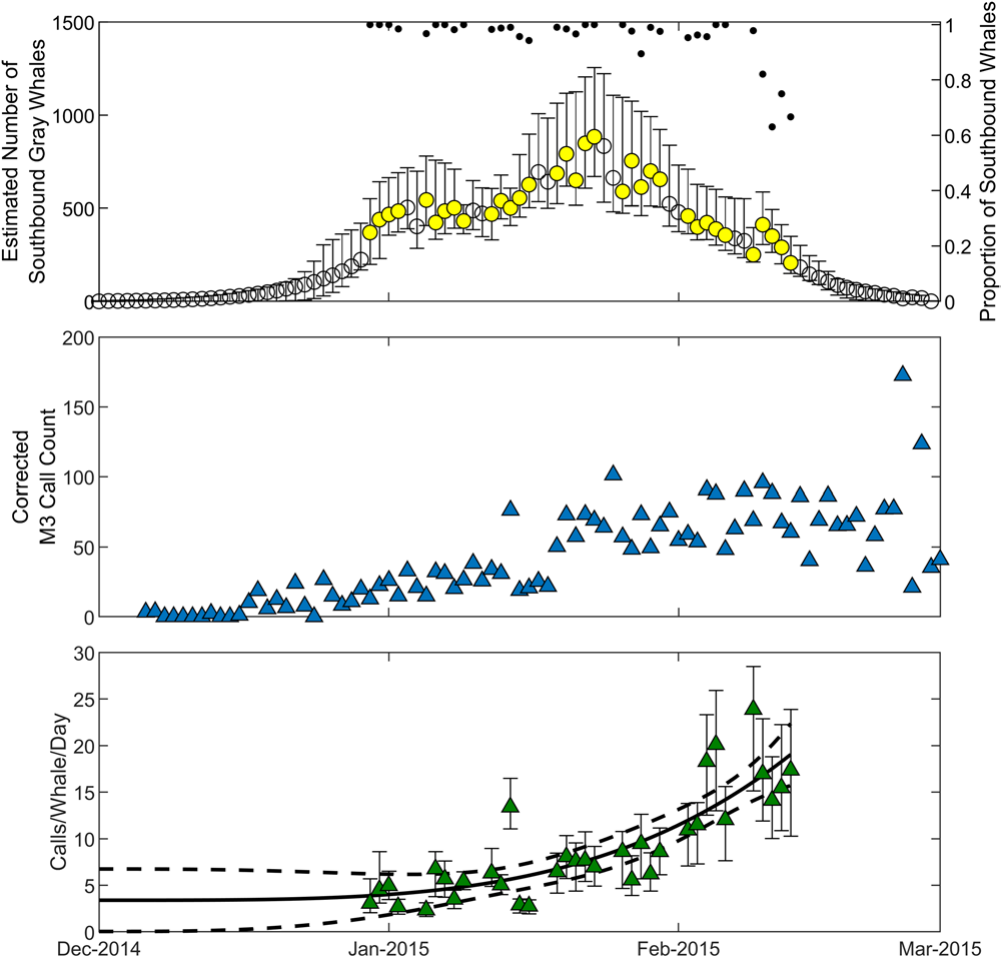
Gray whale acoustic recordings and visual sightings. The top plot shows the modelled number of southbound gray whales that passed Granite Canyon each day based on visual sightings (circles, left axis) and the proportion of gray whales that were travelling south (dots, right axis). Yellow circles indicate days when visual observers were on-effort. Error bars show the 95% Credibility Interval of the modelled abundance estimates. The middle plot shows the corrected M3 call count in blue triangles (corrected based on probability of localization). The bottom plot shows the daily calling rate based on the number of M3 calls and the number of estimated whales for days when visual observers were on-effort. The solid line is the two-term power model with the dashed line showing the 95% non-simultaneous prediction interval for the calling rate function. The coefficients of the two-term power model are *a* = 1.22 × 1.0^−6^, *b* = 3.79, and *c* = 3.39. The error bars show the uncertainty in the calling rate based on the 95% Credibility Interval of daily gray whale abundance estimates.

The daily calling rate increased over the southbound migration from 30 December 2014 until 13 February 2015 (Fig. 3). Calling rate ranged from 2.3 calls/whale/day on 5 January to 24.0 calls/whale/day on 9 February. We fit a two-term power model to the daily calling rate estimates on days when both visual and acoustic data were collected. Using the acoustic call counts from 6 December, when calls began, until 29 December and the extrapolated calling rate from the two-term power model, we estimated that 4,340 whales passed Granite Canyon prior to the onset of visual observations on 30 December 2014. Since calling rate is inversely related to number of whales, using the upper bound of the 95% non-simultaneous prediction interval of the calling rate function to estimate the minimum number of whales resulted in a minimum of 2,418 whales that passed between 6–29 December. The prediction interval was wider during these dates in December because there was no calling rate data. The lower bound of the 95% prediction interval during this time approached zero, which, when used in the denominator to estimate the maximum number of whales, resulted in a maximum of 38,304 whales.

### Infrared camera detections and visual sightings

**Figure 4:**
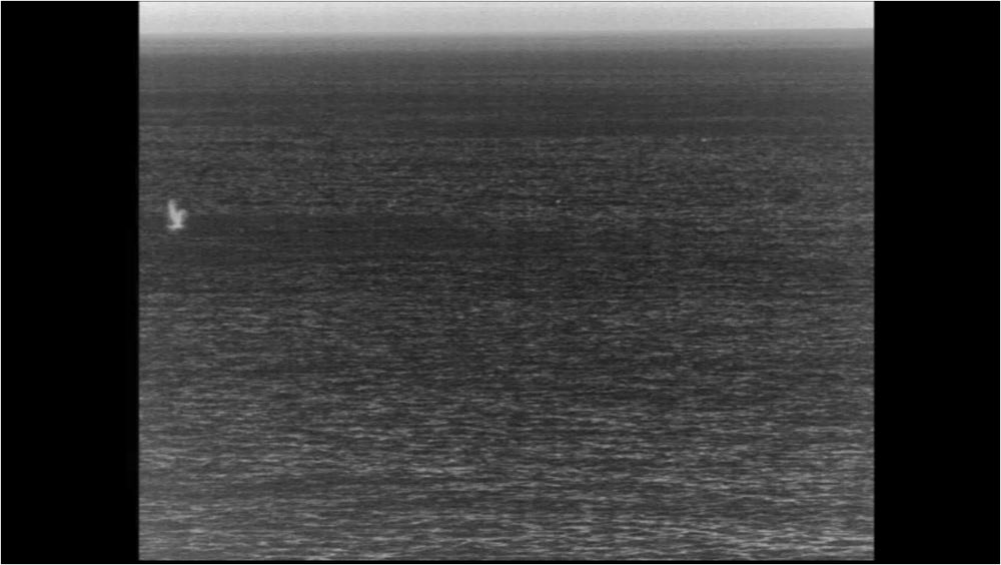
Example frame showing a gray whale blow recorded on the infrared camera on 05 Jan 2015.

**Figure 5:**
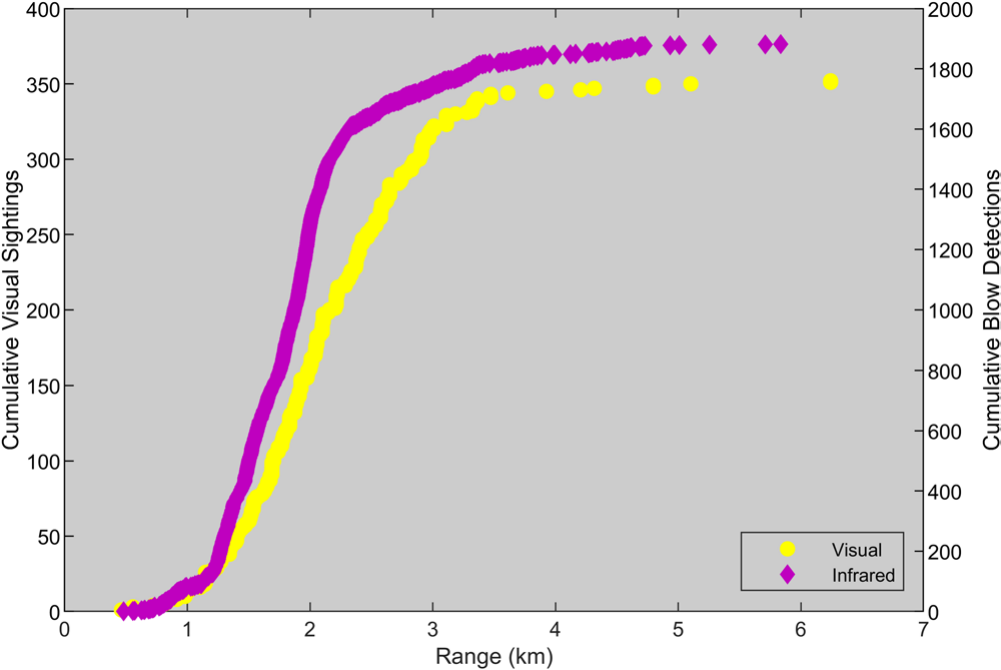
Cumulative visual and infrared detections as a function of range. Cumulative visual sightings (yellow circles and left y-axis) for 5–8 January 2015 increase at a relatively constant rate until a range of approximately 3 km, while cumulative infrared blow detections (pink diamonds and right y-axis) increase at a constant rate until a range of approximately 2.1 km. Visual sightings do not have a probability of detection that decreases with range (confirmed with aerial sightings), but the probability of detection for blows decreases after 2.1 km.

Blows were detected using the shore-based infrared (IR) camera between 479 m and 5.8 km offshore, while observers sighted gray whales between 185 m and 9.4 km offshore. An example frame with a blow is shown in Fig. 4. In order to determine the search area with reliable infrared blow detection, we compared the cumulative distribution of infrared blow detections and gray whale sightings as functions of distance offshore. The distribution of gray whale sightings was representative of the true distribution of gray whales as verified by aerial surveys^16^. We plotted cumulative number of blows detected using the camera and cumulative number of sightings on the same days as functions of range (Fig. 5). The cumulative number of blows versus range had a shallower slope indicating a lower density of blows from the closest detectable distance to 1.2 km range offshore and then a steeper slope indicating a higher density of blows from 1.2 km to 2.1 km range offshore. Beyond this range, few blows were detected and the slope decreased as the cumulative number of blows approached the total blow count. In contrast, the same plot of cumulative visual sightings had a shallower slope that transitioned into a steeper slope until about 3 km range, and then the slope decreased again due to the decreasing density of whales at farther distances from shore, which was expected for the nearshore gray whale migration. Since the range at which the density of IR camera blows decreased was less than the range at which the true density of whales from shore decreased, we can conclude that we were unable to reliably detect gray whale blows beyond 2.1 km offshore. Therefore, we set the probability of detection for the IR camera to 100% for ranges less than or equal to 2.1 km and only used blows and animal abundances within this range to estimate blow rate. In addition, we did not use detections closer than 500 m due to the very narrow field of view as the range offshore approached zero. Based on the distribution of visual sightings on 5–8 January, we estimated that 53% of the gray whales that migrated south on these days, were between 500 m and 2.1 km from shore.

The probability of IR blow detection was the same at night and day. The number of blows during the 9-hour visual observer watch (07:30–16:30) was approximately 9/24 of the number of blows detected over the full day, which is what was expected if the probability of blow detection by the camera did not change between night and day. In addition, the Wilcoxon rank sum test did not reject the null hypothesis that the median number of blows per hour were equal when comparing hours in the night with hours in the day (p = 0.55), further supporting the observation that the probability of blow detection was the same at night and day.

In total, 1,427 blows were detected with the IR camera between 0.5 to 2.1 km offshore on 5–8 January 2015. These blows were corrected for the proportion of the day with IR camera video assuming 100% probability of detection when video was present. The corrected number of blows was 1,447 over these four days. Since the mean swimming speed of migrating gray whales is 1.6 m/s, the mean travel time through the 6.2° camera field of view was 1.7 min. Using the proportion of gray whales sighted between 0.5 and 2.1 km from shore, the estimated number of whales within that range over these four days was 1,041. The average blow rate was therefore 49 blows/whale/hour on 5–8 January 2015 (using the 95% Credibility Interval for gray whale abundance: 25.5–74.9 blows/whale/hour). Results from all four days are summarised in Table 1.

**Table 1:** Summary of infrared blow detections with ranges greater than 0.5 km and less than or equal to 2.1 km.

Date	Detected Blows	Corrected Blows	Median Whales	Whales Within Range	Blow Rate (Blows/Whale/Hour)	Uncertainty Bounds (Blows/Whale/Hour)
05 Jan 2015	435	442	544	290	53.5	37.3–71.5
06 Jan 2015	291	294	423	225	45.7	25.5–58.1
07 Jan 2015	407	413	484	258	56.0	36.5–74.9
08 Jan 2015	294	298	502	268	39.1	27.7–49.9

Detected Blows are the total number of whale blows manually detected and Corrected Blows increases the number detected by the fraction of the day with no infrared video assuming 100% probability of detection in this interval of ranges. Median Whales are the modelled median number of whales estimated to migrate south past Granite Canyon from visual sightings. Whales Within Range are the estimated number of those whales that are travelling within 0.5 to 2.1 km from shore based on the offshore distribution from visual sightings. Blow Rate is calculated by dividing the corrected number of blows by the number of whales within the IR camera search area and by the average amount of time it takes for a whale to travel through that search area. Uncertainty Bounds show the blow rates based on the 95% Credibility Interval of daily gray whale abundance estimates.

## Discussion

This study combined visual sightings, acoustic call detections, and infrared camera blow detections to calculate the cue rates of migrating eastern North Pacific gray whales. Cue rates are needed to estimate abundance of animals not observed directly. This paper is a first step to utilizing cue rates to calculate abundance of marine mammals. Ultimately, these techniques can be applied to other marine mammals to monitor their abundance over long time periods.

Using visual sightings to model the distribution of gray whales from shore allowed us to document the number of animals in the study area with greater accuracy than in a previous study^8^. We estimated that the average calling rate over the entire migration season was 7.5 calls/whale/day, very similar to the 7.9 calls/whale/day calling rate measured by Dalheim^9^ in the Baja California lagoons in February. As is apparent from this result, any changes in the number of animals that were in our search areas as well as probability of detection would shift our results, so it is important to ensure these values are as accurate as possible.

The acoustic recordings indicated that gray whales called at a rate that increased by more than a factor of 5 over the duration of the southbound migration. We hypothesise that the segmented demographic phases of the migration produced this trend. That is, the forerunners of the migration are pregnant females followed then by mature whales and finally juveniles. Juveniles are undertaking their first southbound migration, fully weaned from their mothers, and may call at a higher rate than their mature and more experienced conspecifics. In contrast, Guazzo *et al*.^8^ found that the percentage of calls that were part of an acoustically localised track was high at the start of the southbound migration and then decreased toward the middle of the migration. In order for calls to be grouped into a track, whales had to be calling at a high enough rate to have at least 5 calls localised within the time that they were passing through the array. We hypothesise that a small proportion of early gray whales were calling at a high rate, but the overall calling rate was low at this time, while in the middle of the migration, more whales were calling, but not at a high enough rate to be tracked. This positive trend in calling rate over time has important implications for estimating abundance of whales using acoustic methods. An average calling rate should only be used to estimate the number of animals if the averaging is over the same timescale as the acoustic detections. For instance, to estimate the number of animals present on a given day from number of calls, one must use the average calling rate for that day and not an average calling rate over any longer timescale. Further work should be done to check the predicted trend in calling rate for early segments of the southbound migration and for the northbound migration by increasing visual sighting effort during these times. Additionally, the calling rate should be monitored for multiple years to assess its stability and whether acoustic cue rate is a predictable enough metric to use to estimate abundance.

Acoustic recordings were also used to estimate the number of whales that migrated south before visual observers began their effort on 30 December 2014. Using a two-term power model fit to the daily calling rate values, we extrapolated calling rate and estimated that 4,340 whales migrated south between 1–29 December 2014 (minimum of 2,418 whales using the upper 95% non-simultaneous prediction interval of the calling rate function), which is 2,829 more whales than the sum of the daily median values that NOAA estimated travelled past during that time (NOAA median value: 1,511, 95% Credibility Interval: 483–2,982)^2^, or an increase of approximately 10% of the estimated population. This increase in whale abundance is within the 95% Credibility Interval reported for the gray whale abundance for the entire migration season, but is greater than the 95% Credibility Interval for 1–29 December, however there is overlap between the two intervals. It is important to note that the 2014–2015 gray whale population size model fit the visuals data poorly^2^. Therefore, we do not think it is appropriate to add our estimate of additional whales during December to the full population size value to get a new population size. However, these results show how cue rates and multiple detection methods can reduce the uncertainty in modelled abundance values derived from a single method. Given the abundance of acoustic calls detected during the early part of the southbound migration (i.e. December) and the greater estimated number of whales reported here than in previous estimates during this time, NOAA will begin its gray whale visual surveys at the beginning of December in 2019 and 2020 to better document whale numbers during this previously under-sampled period of the migration.

Infrared cameras have great potential to monitor as effectively at night as during the day and for longer durations than possible for visual observers. The blow rates we measured over the selected four days (49 blows/whale/hour) were similar to those reported by Sumich^14^ from visual observations (43.2 blows/whale/hour) and the range of blow rates over the four days (39–56 blows/whale/hour) was well within Sumich’s measured range (27–67.2 blows/whale/hour). However, the infrared camera had a decreased probability of detecting blows at ranges beyond 2.1 km. Visual observers use handheld binoculars to search for whales, but detecting distant blows from a video is difficult due to decreasing image resolution as range increases. One solution to this problem would be to have multiple cameras optimised to monitor different ranges offshore. In addition an accurate automatic detector system needs to be created to detect blows so that: (1) blow rate can be calculated over the entire migration and (2) a more accurate cue rate can be calculated that can be used to estimate gray whale abundance. We found that a detector system created by Toyon Research Corporation did not perform up to the necessary standards since multiple runs of the detector resulted in different numbers of detections. Zitterbart *et al*.^17^ developed an automated infrared detector for a ship-based camera so perhaps something similar could be used for the land-based camera. To calculate probability of detection, it is necessary to compare number of blows detected with the true number of blows produced. Since we did not have a value for the number of blows produced, we assumed that we detected 100% of the blows produced within the constrained search area during good visibility conditions. Previous studies have used number blows counted by visual observers and have assumed 100% probability of detection for these human observers. However, neither of these assumptions are probably accurate. An experiment or simulation could be used to calculate the true probability of detection of whale blows with these autonomous methods. Finally, we chose times when the environmental conditions were ideal to monitor for infrared detections. Effort should be invested to estimate the probability of detection when conditions are not ideal for infrared detection, such as days with high humidity, haze, or choppy seas.

For any observation method, cue rates that approach zero will lead to animal abundance estimates that approach infinity with high levels of uncertainty. The lower 95% bound of the calling rate prediction interval is an example. Very low cue rates should be approached with caution, similarly to how abundance estimates from visual surveys should be treated when there is a very low probability of detecting a whale. In this case, we are able to combine methods and use knowledge of the gray whale population to conclude that this upper value for the number of gray whales is unrealistic. However, before a single method is used to detect whales and estimate abundance, cue rate should be validated over multiple years and estimated number of whales should be checked between methods in order to constrain the prediction intervals.

Unfortunately, Durban *et al*.^2^ reported that the 2014–2015 gray whale abundance model did not fit the visual sightings data well. Right-skewed daily abundance distributions pulled the abundance estimations higher and resulted in large probability intervals. We calculated the upper and lower bounds of cue rates using the 95% Credibility Interval of the daily abundance values, but we recommend verifying cue rates across multiple years before substituting cue detection methods for visual observations to estimate abundance.

Cue rates can help estimate animal abundance from autonomous detections. Autonomous detections of marine mammals are more predictable than human observations, can operate consistently over longer periods of time, and may be especially advantageous in environments where human observations are not possible. For cetaceans, acoustic or infrared detections seem most viable. In this study, we combined acoustic call recordings and infrared blow detections with visual sightings to calculate cue rates of migrating gray whales. Acoustics-based approaches are advantageous in poor visibility conditions, but infrared blow rates are likely more stable over time than calling rates since they are less dependent on behaviour. Acoustic receivers and infrared cameras have the benefit of being able to monitor at all times of the day and these methods can be automated so that detection algorithm performance can be quantified objectively. However, further work is needed before cue rates and autonomous sensing methods will be ready to substitute for visual observers. Cue rates need to be monitored over long periods of time to determine their stability and predictability. Probability of detection needs to be better understood, especially the probability of detecting blows with IR cameras in various conditions. Detectors need to be developed to automatically detect the cue of interest so that it is possible to analyse continuous, long-duration recordings. Whenever possible, visual surveys should be paired with acoustic and/or IR recordings to increase the sample size used to calculate cue rates and our understanding of how the cue rates change with time and location. Even with this necessary initial effort, infrared detectors and acoustic sensors have the potential of greatly improving marine mammal abundance estimates.

## Methods

We combined three methods of sensing and observing gray whales. Visual observers counted whales as they migrated past the study site, hydrophones recorded the whale acoustic calls, and infrared cameras detected the true or apparent temperature difference between the whale blows and the surrounding water. The observation site was at the NOAA Granite Canyon facility located on Big Sur coast in central California. The continental shelf is about 4 km wide at this location, so it is an ideal place to census gray whales since they stay close to shore to migrate over the shelf. Figure 1 shows a map of the survey area.

### Visual sightings

Visual observers from the National Oceanic and Atmospheric Administration (NOAA) counted southbound gray whales from the shore at Granite Canyon during acceptable weather conditions for 34 days from 30 December 2014 to 13 February 2015. The visual observers were 22.3 m above sea level at 36°26′ 23.61″N 121°55′20.52″W. Observers worked in pairs and counted whales using the naked eye and 7 × 50 binoculars. For each sighting of a group of gray whales, observers recorded the date, time, bearing, distance, group size, direction of travel, Beaufort Sea State, and visibility. The bearing angles had an error +/−1° due to the potential influence of the metal visual observation shelter on the observer compasses. Full visual methods are provided by Durban *et al*.^18^. Raw counts were corrected for the estimated probability of visual detection and the estimated number of whales that passed when the visual observers were off-effort. A Bayesian model was used to estimate the number of southbound gray whales that migrated past per day over 90 days starting on 1 December. Durban *et al*.^18^ described the model used to estimate gray whale abundance from visual counts. Unfortunately the model for this migration season had large probability intervals. We calculate uncertainty bounds for cue rates using the 95% Credibility Interval of the estimated daily abundance of gray whales which was reported by Durban *et al*.^2^.

The offshore distribution of gray whales was calculated from visual sightings. For every southbound pod sighted in visibility and Beaufort conditions less than 5, we assumed the group size and direction were the last values recorded and we estimated the distance offshore using the mean range for all sightings of that pod. The offshore distribution of gray whales from the visual sightings closely matched the offshore distribution from past aerial surveys^16^ (although the aerial surveys were published in more coarse offshore distance intervals), so we concluded that the visual observers did not miss a greater proportion of whales at far distances.

### Acoustic recordings

Four bottom-moored hydrophones continuously recorded sounds offshore of the NOAA SWFSC visual and infrared survey site from November 2014 until June 2015. This work was completed under the Monterey Bay National Marine Sanctuary Research permit MBNMS-2014-039. Table 1 of Guazzo *et al*.^8^ lists locations and depths of the hydrophones. A Generalized Power Law Detector (GPL)^19^ detected potential calls in the acoustic data from each of the four hydrophones. All detections were manually verified to be gray whale calls by inspecting spectrograms across the four channels. Detections were localised using the time difference of arrival of the call on each hydrophone compared to the NE hydrophone. Raw call counts were corrected for the estimated probability of detection and localization within the search area based on acoustic propagation properties and background noise. Guazzo *et al*.^8^ presented a full description of the acoustic methods.

Calling rate was calculated by combining modelled daily whale abundances from visual sightings^2^ with acoustic call counts. We did not correct the modelled abundance values for the previously assumed 8% difference in nighttime passage rate since we did not observe a diel change in mean speed or direction for acoustically tracked gray whales^8^. To calculate calling rate, the corrected call count was divided by the estimated number of whales that migrated through the hydrophone array and the time that they spent within the area of the array. We estimated that 68% of the whales migrated through the array instead of the 90% value we previously assumed^8^. This value of 68% was based on the offshore distribution of whales from the visual sightings during this same migration season. The previous assumption of 90% was based on aerial sightings data from other migration seasons^16^. These aerial sightings were published in coarse increments of distance offshore, but using visual sightings data from the 2014–2015 migration season allowed us to use precise estimates of the proportion of whales that migrated through the search area in this study. The time that a whale spent in the array was approximately 23.75 min, based on the mean measured swimming speed of acoustically tracked animals of 1.6 m/s and the distance through the array of 2.28 km^8^. The calling rate was estimated every day that visual observers were on-effort. Because visual observers only included southbound whales in their daily estimates but calls may be produced by whales travelling southbound or northbound, the proportion of visually sighted whales that were travelling southbound was multiplied with the corrected M3 call count to estimate the number of these calls that were produced by southbound whales. The percentage of southbound whales was above 80% until the last three sighting days when the percentage decreased to between 63–75% (Fig. 3). This method assumes that southbound and northbound whales are calling at the same rate. The calling rate calculations are explained in more detail in the Cue Rate Formulas section.

We used the calling rate calculated from days with both visual observations and acoustic recordings to calculate the number of gray whales that migrated south before the visual survey effort began. A two-term power model of the form *f*(*x*) = *ax*^*b*^ + *c* was fit to the daily calling rates and extrapolated back to 1 December (x = 1). This model was selected because the extrapolated calling rate remained relatively constant for the days prior to the visual census start date, which we decided was the most realistic assumption during this time period. If the calling rate alternatively approached zero, the estimated number of whales would increase to approach infinity. In addition, the two-term power model had a low summed square of residuals (SSE) when fit to the calling rate data. To calculate the number of animals that migrated past Granite Canyon on 1–29 December 2014, we divided the estimated number of calls localised within the area of the array on each day by the extrapolated daily calling rate, the estimated proportion of animals within the array, and the time spent in the array (see Cue Rate Formulas). We calculated the 95% prediction interval using the non-simultaneous (pointwise) prediction interval for the calling rate function.

### Infrared camera detections

Three infrared cameras pointing at different angles offshore recorded video from December 2014 until June 2015. The infrared cameras were located about 41 m from the visual observers at 36° 26′24.61″N 121°55′19.41″W. For the analysis in this paper, we used the data from the middle camera on 5–8 January 2015. These four days had simultaneous visual, acoustic, and infrared observations during good weather conditions with little wind. The centre of the lens of the middle camera was 28.1 m above sea level. The camera was an FLIR F-606 model with a lens focal length of 100 mm and 640 × 480 pixel resolution (http://www.flir.com/security/display/?id=44258). The camera had a long-life, uncooled VOx microbolometer detector which sensed radiation with wavelengths of 7.5 to 13.5 *μ*m. The field of view was 6.2° in the horizontal direction and 5° in the vertical direction. The video had a sample rate of approximately 30 frames/s. These images were manually processed using software in which an analyst could watch the video and click on the location of each blow. The software is available on GITHUB (https://github.com/rguazzo/Whale-Blow-Logger). The pixel locations of the blow were converted into latitude and longitude by calculating range and azimuth. Range was computed by finding the angle of the blow from the horizon using linear interpolation of the vertical field of view of the camera and then using the geometry and equations detailed by Gordon^20^ to calculate the distance in meters. Azimuth of the blow was calculated by linear interpolation from the known angles of the edges of the video to the blow.

In theory, the probability of detection for an infrared camera could be estimated in a similar way as probability of detection for an acoustic system. This probability is affected by the source characteristics or the temperature, size, and shape of a gray whale blow; the noise characteristics or glare from the sun and waves on the ocean; and the transmission loss which is affected by carbon dioxide and humidity since these molecules absorb infrared wavelengths^13^. During the four days that the infrared camera detections were analysed, visual observers described calm seas so noise from waves was not a factor. However, accurate physics-based infrared propagation modelling was not possible since we did not have simultaneous sensors over the ocean measuring the aerosol content of the air. Instead, the colour of the video frames was used to measure visibility. The RGB matrix of each video frame was converted into greyscale and the average colour value for each row of pixels was calculated over the length of each video. The ability to detect a whale blow depended on the contrast between the light blow and the dark water which was quantified as the colour variance across the vertical axis. During times of lower visibility, the water had a lighter colour in the IR image and the variance across the vertical axis of the video frame was lower. We measured blows per second as a function of colour variance across the vertical axis of the video frame to assess if the observed changes in variance affected the detectability of blows. The colour variance was not a function of the number of blows detected, so we concluded that for these days, probability of detection was not impacted by visibility.

Probability of detection of a gray whale blow using the IR camera was, however, impacted by distance offshore. We plotted the cumulative number of blows versus range to evaluate the impact of range on detectability (Fig. 5). If, for example, whales were randomly distributed and every blow was detected, the function should be proportional to the search area with a positive, increasing slope equal to the density of blows (blows/km range). The probability of detection of visual sightings did not decrease with range (based on comparison with past aerial surveys^16^), so the shape of the cumulative sightings versus range was representative of the true offshore distribution of gray whales. Since we compared the distribution of infrared detections at night and day with visual detections during the day, this method assumes whales were not changing their distribution at nighttime, which we confirmed with acoustically tracked gray whales^8^. We compared the shape of the plots of the cumulative number of sightings of whales and detections of blows. In particular, we focused on the ranges at which the slopes of the cumulative IR camera blow detections and visual sightings decreased indicating a decrease in density after these ranges. If the slope of the IR blow detections decreased at a closer range than that of the visual sightings, then the density of the blows detected decreased closer to shore than the true density of whales, and therefore the probability of detection of blows with the IR camera decreased beyond this range offshore.

Gray whale blow rate was calculated in a similar way as call rate. The number of blows within the search area was divided by the number of whales estimated to migrate through that search area and the average time it takes to travel through the area. We used the visually-determined distribution of whales on the same four days as the infrared camera videos to estimate the proportion of whales within the search area. To calculate the time spent in the search area, we used the range of sighted whales and calculated the arc length within the camera field of view. We divided the average arc length by the average swimming speed calculated by Guazzo *et al*.^8^ to estimate average time within the field of view. The Cue Rates Formulas section presents the equations used in these calculations in detail.

### Cue rate formulas

Cue rate (for both call rate and blow rate) was estimated as

1 $$\hat{r}(t)=\frac{{\hat{N}}_{C}(t)}{{\hat{N}}_{W}(t)\,{\hat{P}}_{SA}\,{\hat{\bar{t}}}_{SA}}$$

where ${\hat{N}}_{C}(t)$ is the estimated number of cues that occur in the search area as a function of time (here we usually used 1 day as the time period), ${\hat{N}}_{W}(t)$ is the estimated number of whales present from the abundance model as a function of time, ${\hat{P}}_{SA}$ is the estimated proportion of whales that are in the search area based on visual sightings, and ${\hat{\bar{t}}}_{SA}$ is the estimated average amount of time a whale spends in the search area. The units for cue rate are cues/whale/unit time.

The number of cues ${\hat{N}}_{C}(t)$ was estimated using the number of raw cues detected and the probability of detection. For acoustic calls and using nomenclature very similar to that used by Marques *et al*.^3^

2 $${\hat{N}}_{C}(t)={n}_{c}(t)\frac{1-{\hat{c}}_{N}(t)}{{\hat{P}}_{L}(t)\,(1-{P}_{NE})}$$

where *n*_*c*_(*t*) is the number of raw calls detected and localised, ${\hat{c}}_{N}(t)$ is the estimated proportion of northbound and milling whales, ${\hat{P}}_{L}(t)$ is the estimated probability of localization, and *P*_*NE*_ is the proportion of time with no effort. Since only southbound whales were included in the visually estimated daily abundance, calls from whales swimming northbound or milling represent false alarms. The estimated proportion of northbound and milling whales, ${\hat{c}}_{N}(t)$, was determined from visual records of the bearing of all sighted gray whales. Guazzo *et al*.^8^ described the methods used to estimate probability of localization ${\hat{P}}_{L}(t)$. To summarise, 100 source level realizations were randomly selected from the probability distribution of source levels estimated in the paper. For each of these source levels (*SL*_*dB*_), transmission loss (*TL*_*dB*_) was calculated across an (x,y) grid of source locations to each hydrophone^8^, noise level (*NL*_*dB*_) was calculated for each hydrophone for each minute of recording, and the received signal to noise ratio (*SNR*_*dB*_) was calculated for the i-th hydrophone as

3 $$SN{R}_{dB}(x,y,i,t)=S{L}_{dB}-T{L}_{dB}(x,y,i)-N{L}_{dB}(i,t)$$

where source level, transmission loss, and noise level are calculated in units of dB re l*μ*Pa at 1 m, dB of received level at range given by (x, y) relative to received level at 1 m, and dB re l*μ*Pa over the frequency band of interest (20–100 Hz), respectively. We took the minimum SNR across the four hydrophones and calculated the proportion of the 100 source level realizations with SNRs greater than the 0.5 dB detection threshold to get the estimated probability of localization at that source location and time, ${\hat{P}}_{L}(x,y,t)$. We then averaged over the search area to get the estimated probability of localization for that minute, ${\hat{P}}_{L}(t)$. If ${\hat{P}}_{L}(t) < {\rm{0.5}}$, any calls detected in that minute were not included in the total *n*_*c*_(*t*) and that minute had no effort. The proportion of minutes in a day with no effort was *P*_*NE*_.

Durban *et al*.^2,18^ derived the estimated number of gray whales migrating south past Granite Canyon per day ${\hat{N}}_{W}(t)$. The daily abundance estimates were not corrected for a different nighttime passage rate since acoustic tracks for this migration season indicated the migration rate was the same at night and day^8^.

The average amount of time for a whale to travel through the acoustic search area ${\hat{\bar{t}}}_{SA}$ was estimated by dividing the average distance travelled through the search area by 1.6 m/s, the mean speed estimated from acoustic tracks^8^. For the acoustic recordings, ${\hat{\bar{t}}}_{SA}=23.75$ min^8^.

Similarly, the number of estimated blows was calculated using

4 $${\hat{N}}_{C}(t)={n}_{b}(t)\frac{1-\hat{c}(t)}{{\hat{P}}_{D}(t)\,(1-{P}_{NE})}$$

where *n*_*b*_(*t*) is the raw number of blows detected, ${\hat{P}}_{D}(t)$ is the estimated probability of detection, and as before, *P*_*NE*_ is the proportion of time with no effort. Since the blows were detected in the infrared video by a human operator and these four days with infrared detections were well before the northbound migration, the probability of false detection, $\hat{c}(t)$, was zero. Also, for whales with ranges ≤2.1 km, ${\hat{P}}_{D}(t)=1$. No blow detections less than 500 m or farther than 2.1 km were included in *n*_*b*_(*t*). Short time gaps existed between adjacent video files, resulting in a total proportion of minutes with no video data, *P*_*NE*_. The average amount of time for a whale to travel through the IR search area ${\bar{t}}_{SA}$ was estimated using the proportion of visually sighted whales at each range (r), ${n}_{W}({r}_{j})/\sum _{j}\,{n}_{W}({r}_{j})$, and the IR camera’s 6.2° horizontal field of view

5 $${\widehat{\bar{t}}}_{SA}=2\pi \frac{{6.2}^{\circ }}{{360}^{\circ }}\sum _{j}\,(\frac{{n}_{W}({r}_{j})}{{\sum }_{j}\,{n}_{W}({r}_{j})}\,\frac{{r}_{j}}{s})$$

where s = 1.6 m/s and j is the index for each observed range value. For the IR camera recordings, ${\hat{\bar{t}}}_{SA}=1.7$ min.

To use the number of cues to calculate the number of whales, we can solve Eq. 1 for ${\hat{N}}_{W}(t)$. This equation was used to estimate the number of whales that migrated past Granite Canyon on 1–29 December 2014 when only acoustic detections were available. Cue rate (calling rate in this case) was extrapolated from data from later in the season when both visual observations and acoustic detections were present.

6 $${\hat{N}}_{W}(t)=\frac{{\hat{N}}_{C}(t)}{\hat{r}(t)\,{\hat{P}}_{SA}\,{\hat{\bar{t}}}_{SA}}$$

### Accession codes

The acoustic dataset is available at http://seamap.env.duke.edu/dataset/1541. The shore-based visual sightings are available at http://seamap.env.duke.edu/dataset/1862. The shore-based infrared camera blow detections are available at http://seamap.env.duke.edu/dataset/1864.
